# Complete RPE and outer retinal atrophy in patients receiving anti-VEGF treatment for neovascular age-related macular degeneration

**DOI:** 10.1371/journal.pone.0232353

**Published:** 2020-05-05

**Authors:** Victor A. Eng, Nadim Rayess, Huy V. Nguyen, Theodore Leng

**Affiliations:** Byers Eye Institute at Stanford, Stanford University School of Medicine, Palo Alto, California, United States of America; University of Florida, UNITED STATES

## Abstract

**Importance:**

Neovascular age-related macular degeneration (nAMD) is a leading cause of blindness with several intravitreal anti-vascular endothelial growth factor (anti-VEGF) agents available for its management such as aflibercept, bevacizumab, and ranibizumab. However, direct comparisons between these three agents among the same patient population are limited.

**Objective:**

To assess the rate and growth of complete retinal pigment epithelium and outer retinal atrophy (cRORA) in eyes with nAMD treated with aflibercept, bevacizumab, and/or ranibizumab.

**Method:**

Retrospective cohort study of patients with treatment-naïve neovascular AMD seen at an academic hospital between October 2006 and February 2019. Study eyes were treated with intravitreal injections of aflibercept, bevacizumab, and/or ranibizumab and followed for two years.

**Main outcomes and measures:**

cRORA prevalence, location, size, and growth rate. Eyes were imaged with Cirrus spectral domain optical coherence tomography (SD-OCT). Presence and size of cRORA were calculated using the FDA-approved Advanced RPE Analysis software. Linear regression models were used to correlate cRORA progression with baseline demographic and ocular characteristics, anti-VEGF drug, and number of injections. Unpaired t-tests, ANOVA, and linear regression models were computed with SAS 9.4.

**Results:**

197 eyes from 158 patients (mean age 78.9, 62.9% women) received an average of 13 anti-VEGF injections over 24 months. 22% developed new cRORA. Mean cRORA area increased from 1.71 mm^2^ to 2.93 mm^2^. At 24 months, eyes with 11+ injections had significantly less cRORA area (11+ injections, 4.02 mm^2^; ≤ 10 injections, 2.46 mm^2^; p = 0.01) and growth rate (11+ injections, 0.41 mm^2^/year; ≤ 10 injections, 1.05 mm^2^/year; p = 0.02). Choice of anti-VEGF drug yielded no significant difference in cRORA progression.

**Conclusions and relevance:**

Treating nAMD with aflibercept, bevacizumab or ranibizumab demonstrated comparable cRORA development at 24 months. Number of injections inversely correlated with cRORA area and growth. These results warrant further investigation in the pathophysiology of cRORA in anti-VEGF treated eyes.

## Introduction

Age-related macular degeneration (AMD) is the leading cause of blindness in industrialized countries with a reported 1.47% prevalence and 1.75 million people affected in the United States alone[1,2]. More than 80% of all AMD cases manifest with the presence of macular drusen without choroidal neovascularization (CNV)[3]. Over time, approximately 15% of patients with non-exudative AMD progress to advanced AMD which can be categorized into two forms: neovascular AMD (nAMD) characterized by CNV, or atrophic AMD characterized by complete RPE and outer retinal atrophy (cRORA)[4]. While nAMD is the more common of the two, both forms are associated with severe vision loss[5].

A number of intravitreal injection agents that inhibit VEGF are currently used to limit the progression of neovascular AMD: bevacizumab, ranibizumab, and aflibercept. Bevacizumab and ranibizumab are closely-related recombinant humanized monoclonal antibodies that bind to VEGF. Bevacizumab is a full-length antibody while ranibizumab is an Fab fragment of the same antibody precursor[6,7]. Ranibizumab has a higher binding affinity than bevacizumab, and has been approved by the FDA for use in neovascular AMD[6]. Bevacizumab—although not currently FDA-approved for AMD—is used in an off-label fashion because it is considerably more cost-effective[8]. Aflibercept, a recombinant fusion protein that acts as a decoy receptor for VEGF, is another anti-VEGF therapy approved for the treatment of nAMD[9]. In separate studies, bevacizumab and aflibercept are found to be non-inferior to ranibizumab in preserving visual acuity[10,11]. Bevacizumab and aflibercept have not been directly compared.

Adverse effects of intravitreal anti-VEGF injections include infectious endophthalmitis, retinal detachment, ocular hemorrhage, and others[12]. Treatment of nAMD with anti-VEGF injections has also been observed to increase the risk of RPE atrophy as seen in atrophic AMD[13–15]. Anti-VEGF agents lower the amount of soluble RPE-derived VEGF isoforms that appear necessary for the maintenance of the choroid. The absence of soluble VEGF in mice experiments promoted drusen accumulation and barrier dysfunction, resulting in loss of RPE and underlying choriocapillaris[16]. This pathophysiology and vision loss from subsequent death of overlying photoreceptors closely recapitulates the disease progression of atrophic AMD. Comparative analysis from Comparison of Age-related macular degeneration Treatment (CATT) trial found ranibizumab to cause a significantly higher risk of retinal atrophy development[17]. Smaller studies comparing aflibercept with ranibizumab suggest increased atrophy among aflibercept-treated patients[18–20].

Previous studies that investigated RPE and outer retinal atrophy after anti-VEGF therapy have primarily focused on comparing two agents or have included patients previously treated with another anti-VEGF medication. The current study compares the individual effects of all three major anti-VEGF agents (bevacizumab, ranibizumab, and aflibercept) among patients with no prior history of intravitreal anti-VEGF injections. This study does not include eyes treated with brolucizumab or conbercept, which recently received FDA approval in October 2019 and Chinese FDA (CFDA) approval in November 2013, respectively. Nor does this study include eyes treated with drugs that are currently under investigation (e.g. faricimab, abicipar). The patient population represented at Stanford Byers Eye Institute is ideal for examining this relationship due to the volume of patients as well as the use of all three anti-VEGF agents in clinical practice. Because of the similar visual acuity outcomes across the aforementioned medications, off-label use of bevacizumab is increasingly administered due to its lower cost[8,21]. This study sought to determine whether clinical safety benefits were consistent with considerations in the selection of intravitreal anti-VEGF injections.

## Methods

The study design was approved by the Stanford Institutional Review board and complied with the Health Insurance Portability and Accountability Act regulations, the Declaration of Helsinki and all state and federal laws.

### Study participants

Data was retrospectively collected for patients with nAMD who received injections of intravitreal anti-VEGF therapy with aflibercept (Eylea; Regeneron, Tarrytown, NY), bevacizumab (Avastin; Genentech, South San Francisco, CA), and/or ranibizumab (Lucentis; Genentech, South San Francisco, CA) between October 2006 and February 2019 at Byers Eye Institute at Stanford. Patients were considered eligible if the following criteria were met: 1) follow-up of at least 2 years and 2) optical coherence tomography (OCT) imaging at baseline and 2 years. Patients were excluded for any of the following: 1) previous treatment with intravitreal anti-VEGF injections, intravitreal steroids, intravitreal pegaptanib, photodynamic therapy, laser photocoagulation, or ocular surgery; 2) past or concomitant diagnosis of diabetic macular edema, central/branch retinal vein occlusion, central serous chorioretinopathy, posterior uveitis, or optic neuropathy/atrophy; or 3) OCT imaging performed on a device other than Cirrus HD-OCT. The choice of anti-VEGF treatment was made following joint consensus between physician and patient. All study participants received three monthly loading doses of anti-VEGF injections before transitioning to a treat-and-extend regimen. Eyes were treated at each visit with follow-up visits extended by 2-week intervals if visual acuity was maintained or improved, and if OCT demonstrated an absence of intraretinal and/or subretinal fluid. Duration between injections was shortened by 2-week intervals if visual acuity declined or if intraretinal or subretinal fluid persisted or increased. Patients were switched to an alternative anti-VEGF drug if visual acuity or subretinal fluid failed to respond after 3 consecutive injections.

### Study outcomes

Demographic data collected at baseline include candidate risk factors for complete RPE and outer retinal atrophy such as age, sex, BMI, smoking status, use of AREDS supplements or anti-inflammatories, and history of hypertension, hyperlipidemia, diabetes, or cancer. Best-corrected visual acuity (BCVA) and OCT imaging were collected at baseline and 1, 3, 6, 12, and 24 months follow-up. For statistical analysis, Snellen BCVA was converted to the equivalent logMAR by converting to decimal acuity and taking the negative logarithm[22]. Choice and frequency of anti-VEGF injection was recorded throughout the follow-up period. The primary outcome of the study was the change in the location, size, and annual growth rate of incident and prevalent cRORA at each follow-up visit as compared to baseline. The secondary outcome was to identify potential risk factors associated with progression of cRORA.

Spectral-domain OCT images were obtained using the Cirrus HD-OCT (Carl Zeiss Meditec; Dublin, CA) with 512x128 macular cube scans centered on the fovea. A single imaging technician performed and reviewed all scans. OCT images with low signal strength or motion artifacts were discarded and rescanned. Two physicians graded each OCT scan and any discrepancies were resolved with joint discussion. The area of cRORA was quantified using the FDA-approved Advanced RPE Analysis Tool within the Cirrus HD-OCT review software. The Advanced RPE Analysis software automatically identifies and quantifies areas of cRORA as small as 0.1 mm^2^ based on increased illumination below the RPE layer visualized on the Sub-RPE Slab[23]. The algorithm has been shown to have high reproducibility and excellent correlation with manual OCT fundus grading[24].

### Statistical analysis

De-identified patient data were securely stored on the REDCap (Research Electronic Data Capture) electronic database. Baseline characteristics and number of anti-VEGF injections were evaluated by univariate analysis (without adjustment for other covariates) as potential risk factors for cRORA development. Risk factors with a P value < 0.20 were included in a multivariate analysis to assess the independent effect of each predictor. The final multivariate model was created by applying a backward selection procedure that retained only those predictors with P < 0.05. Adjusted mean area and annual growth rate were calculated using the final multivariate linear models. Generalized estimating equations (GEEs) were modeled to account for inter-eye correlations in analyses involving unilateral cases and bilateral cases combined[25]. All statistical tests were two-sided with significance level of p < 0.05. Collected data were analyzed with the SPSS software (version 25).

## Results

A total of 197 eyes from 158 patients were included in this study. Baseline characteristics are shown in Table 1. 62.9% were women and the mean age was 78.9 ± 14.6 years. The mean follow-up duration was 24.0 ± 0.6 months. The mean baseline visual acuity in logMAR was 0.63 ± 0.52 (20/85 Snellen equivalent). At 24 months, the final mean visual acuity remained unchanged at 0.63 ± 0.58 logMAR units (20/85 Snellen equivalent). Study eyes received an average of 12.9 ± 6.0 anti-VEGF injections during the follow-up period. 27 eyes received only bevacizumab, 71 eyes received only aflibercept, and 47 eyes received only ranibuzumab. For eyes that received combination therapy, 27 eyes received bevacizumab + aflibercept, 3 eyes received bevacizumab + ranibizumab, and 16 eyes received aflibercept + ranibizumab. 6 eyes received all three agents (bevacizumab + aflibercept + ranibizumab). 113 eyes (57.4%) had cRORA present at baseline. Of the 82 eyes without baseline cRORA, 44 (52.4%) developed cRORA by the last follow-up. Compared to patients with eyes that did not develop cRORA, patients that did develop cRORA were older (81.3 versus 74.6 years; p = 0.01), had better baseline visual acuity (0.29 versus 0.71 logMAR; p < 0.01), and had marginally longer follow-up (24.1 versus 23.8 months; p = 0.04). Other baseline characteristics did not differ between the two groups.

**Table 1:** Patient demographics.

	All Eyes	No Incident cRORA	Incident cRORA	Unpaired t-test
**Number of Eyes**	197	38	44	
**Age, years**				
** Mean (SD)**	78.92 (14.63)	**76.18 (16.11)**	**81.26 (5.50)**	**p = 0.01**
** Range**	40.65–95.85	47.45–90.46	70.49–94.15	
**Sex, n (%)**				
** Male**	73 (37.06%)	10 (29.41%)	15 (34.09%)	p = 0.67
** Female**	124 (62.94%)	24 (70.59%)	29 (65.91%)	
**Ethnicity**				
** White**	139 (70.56%)	25 (73.53%)	32 (72.73%)	p = 0.94
** Asian**	30 (15.22%)	5 (14.71%)	7 (15.91%)	p = 0.89
** Other**	23 (11.68%)	3 (8.82%)	5 (11.36%)	p = 0.72
**BMI, kg/m^2^**				
** Mean (SD)**	26.44 (5.35)	27.75 (6.15)	25.71 (4.01)	p = 0.15
** Range**	16.49–28.18	17.88–43.94	16.84–34.64	
**Smoking, n (%)**				
** Never**	113 (57.36%)	21 (61.76%)	25 (56.82%)	p = 0.66
** Former**	80 (40.61%)	12 (35.29%)	18 (40.91%)	p = 0.62
** Current**	4 (2.03%)	1 (2.94%)	1 (2.27%)	p = 0.86
**Past Medical History, n (%)**				
** Hypertension**	125 (63.45%)	23 (67.65%)	26 (59.09%)	p = 0.44
** Diabetes**	19 (9.64%)	3 (8.82%)	5 (11.36%)	p = 0.72
** Hyperlipidemia**	89 (45.2%)	17 (50.00%)	17 (38.64%)	p = 0.32
** Myocardial Infarction**	5 (2.53%)	1 (2.94%)	0 (0.00%)	p = 0.26
** Stroke**	7 (3.55%)	0 (0.00%)	1 (2.33%)	p = 0.38
** Cancer**	47 (23.86%)	11 (32.35%)	9 (20.45%)	p = 0.24
**Concurrent Medication, n (%)**				
** NSAID**	58 (29.44%)	12 (35.29%)	13 (29.55%)	p = 0.60
** Steroid (oral)**	16 (8.12%)	5 (14.71%)	2 (4.55%)	p = 0.12
** AREDS Supplement**	135 (68.53%)	21 (61.76%)	27 (61.36%)	p = 0.97
**Duration of follow-up, months**				
** Mean (SD)**	23.95 (0.64)	**23.80 (0.69)**	**24.10 (0.59)**	**p = 0.04**
** Range**	21.59–25.46	21.59–25.30	23.16–25.46	
**Best-corrected visual acuity at baseline, mean (SD)**	0.63 (0.52)	**0.29 (0.23)**	**0.71 (0.48)**	**p < 0.01**
**Best-corrected visual acuity at 2 years, mean (SD)**	0.63 (0.58)	**0.27 (0.39)**	**0.68 (0.61)**	**p < 0.01**
**Atrophy size at baseline, mean (SD), mm^2^**	1.71 (3.40)	0.00 (0.00)	0.00 (0.00)	N/A
**Total anti-VEGF injections, mean (SD)**				
** All participants**	12.93 (5.96)	14.18 (6.57)	14.00 (6.12)	p = 0.90
** Bevacizumab**	9.89 (5.18)	12.00 (0.00)	10.17 (4.40)	p = N/A
** Aflibercept**	14.83 (5.51)	15.42 (5.85)	17.31 (4.99)	p = 0.35
** Ranibizumab**	11.30 (6.00)	10.78 (6.80)	14.86 (5.49)	p = 0.13
** Bevacizumab + Aflibercept**	12.11 (6.16)	14.50 (13.44)	11.33 (7.34)	p = 0.67
** Bevacizumab + Ranibizumab**	11.33 (5.69)	13.00 (0.00)	5.00 (0.00)	p = N/A
** Aflibercept + Ranibizumab**	15.31 (5.12)	19.00 (8.49)	16.00 (1.73)	p = 0.57

Abbreviations: cRORA, complete RPE and outer retinal atrophy; SD, standard deviation; BMI, body mass index; NSAID, non-steroidal anti-inflammatory drug; AREDS, Age-Related Eye Disease Study; anti-VEGF, anti-vascular endothelial growth factor

Risk factors for cRORA development were assessed by univariate analysis of combined data from all 197 study eyes (Table 2). Baseline BCVA (p < 0.01), baseline prevalent cRORA (p < 0.01), baseline cRORA area (p < 0.01), and baseline prevalent drusen (p = 0.02) were all associated with presence of cRORA at the last follow-up. cRORA developing subfovealy correlated positively with baseline BCVA (r = 0.3; p < 0.01) and negatively with cRORA area (r = -0.3; p < 0.01). History of stroke, baseline BCVA, baseline cRORA, and baseline cRORA area were associated with cRORA growth rate after two years. cRORA area was additionally associated with history of hypertension, baseline drusen area, and total number of anti-VEGF injections. On multiple linear regression, only the presence of cRORA at baseline (p < 0.01) and total number of anti-VEGF injections (p = 0.04) were associated with the development of cRORA.

**Table 2:** Univariate regression analysis between potential risk factors and cRORA outcomes at 24 months.

	Presence		Subfoveal Location		Area		Growth	
	R^2^	Significance	R^2^	Significance	R^2^	Significance	R^2^	Significance
**Age**	< 0.01	p = 0.46	< 0.01	p = 0.49	0.01	p = 0.12	< 0.01	p = 0.46
**Sex**	< 0.01	p = 0.88	< 0.01	p = 0.74	0.01	p = 0.10	< 0.01	p = 0.46
**BMI**	< 0.01	p = 0.62	< 0.01	p = 0.87	0.01	p = 0.29	< 0.01	p = 0.18
**Smoking**	< 0.01	p = 0.38	< 0.01	p = 0.63	< 0.01	p = 0.24	< 0.01	p = 0.72
**Hypertension**	< 0.01	p = 0.80	< 0.01	p = 0.26	0.05	**p < 0.01**	0.02	p = 0.06
**Diabetes**	< 0.01	p = 0.35	< 0.01	p = 0.34	< 0.01	p = 0.54	< 0.01	p = 0.67
**Hyperlipidemia**	< 0.01	p = 0.56	< 0.01	p = 0.93	< 0.01	p = 0.31	< 0.01	p = 0.42
**Myocardial Infarction**	< 0.01	p = 0.87	< 0.01	p = 0.36	< 0.01	p = 0.75	< 0.01	p = 0.69
**Stroke**	0.01	p = 0.17	< 0.01	p = 0.98	0.04	**p = 0.01**	0.04	**p = 0.01**
**Cancer**	0.01	p = 0.14	< 0.01	p = 0.98	0.01	p = 0.17	< 0.01	p = 0.48
**NSAID**	< 0.01	p = 0.69	< 0.01	p = 0.86	< 0.01	p = 0.81	< 0.01	p = 0.76
**Steroid**	0.01	p = 0.15	0.01	p = 0.24	0.01	p = 0.31	0.02	p = 0.09
**AREDS**	< 0.01	p = 0.59	0.02	p = 0.16	< 0.01	p = 0.51	< 0.01	p = 0.72
**Baseline BCVA**	0.06	**p < 0.01**	0.07	**p < 0.01**	0.15	**p < 0.01**	0.05	**p < 0.01**
**Baseline Central Subfield Thickness**	< 0.02	p = 0.95	0.01	p = 0.32	< 0.01	p = 0.66	< 0.01	p = 0.93
**Baseline Cube Volume**	< 0.01	p = 0.54	0.01	p = 0.19	0.05	p = 0.15	< 0.01	p = 0.58
**Baseline Cube Thickness**	< 0.01	p = 0.85	0.01	p = 0.28	< 0.01	p = 0.84	< 0.01	p = 0.42
**Baseline cRORA Prevalence**	0.17	**p < 0.01**	< 0.01	p = 0.80	0.05	**p < 0.01**	0.03	**p = 0.02**
**Baseline cRORA Area**	0.05	**p < 0.01**	0.1	**p < 0.01**	0.33	**p < 0.01**	0.13	**p < 0.01**
**Baseline Reticular Pseudodrusen Prevalence**	< 0.01	p = 0.95	0.01	p = 0.32	0.02	p = 0.07	0.01	p = 0.21
**Baseline Drusen Prevalence**	0.03	**p = 0.02**	0.03	p = 0.07	< 0.01	p = 0.49	0.07	**p < 0.01**
**Baseline Drusen Area**	0.02	p = 0.09	0.03	p = 0.06	0.07	**p < 0.01**	< 0.01	p = 0.79
**Baseline Drusen Volume**	< 0.01	p = 0.55	0.02	p = 0.18	< 0.01	p = 0.68	0.01	p = 0.32
**Total Anti-VEGF Injections**	< 0.01	p = 0.65	0.05	p = 0.82	0.04	**p = 0.01**	0.01	p = 0.25
**Number of Bevacizumab injections**	0.01	p = 0.10	0.01	p = 0.31	< 0.01	p = 0.56	< 0.01	p = 0.78
**Number of Aflibercept injections**	0.02	p = 0.06	< 0.01	p = 0.57	0.01	p = 0.13	0.01	p = 0.22
**Number of Ranibizumab injections**	< 0.01	p = 0.45	< 0.01	p = 0.82	0.01	p = 0.33	< 0.01	p = 0.80

Abbreviations: BMI, body mass index; NSAID, non-steroidal anti-inflammatory drug; AREDS, Age-Related Eye Disease Study; BCVA, best-corrected visual acuity; cRORA, complete RPE and outer retinal atrophy, anti-VEGF, anti-vascular endothelial growth factor

Eyes were stratified by anti-VEGF treatment, number of injections, or the development of cRORA. Number of eyes with cRORA, number of eyes with subfoveal involvement, cRORA area, and cRORA annual growth rate is summarized for each visit in Table 3. 144 eyes (73.1%) had cRORA present two years after starting anti-VEGF treatment. 15 eyes with baseline cRORA had no cRORA measurable at 24 months. However, software segmentation error may have been involved given that the majority were small (0.1 mm^2^) at baseline, and manual review of the corresponding OCTs did not find any disappearance of baseline cRORA. Approximately one-third of cRORA cases presented with subfoveal involvement. Mean area of cRORA was 2.93 ± 3.86 mm^2^ and the mean growth rate was 0.60 ± 1.69 mm^2^/year. There was no difference in cRORA prevalence, location, area, or growth rate based on choice of anti-VEGF treatment. On a 3-month treat-and-extend regimen (3 consecutive monthly loading doses + 7 doses over the remaining 21 months), eyes that received more than 10 injections had significantly less cRORA area (2.46 vs. 4.02 mm^2^; p = 0.01) and a significantly slower growth rate (0.41 vs. 1.05 mm^2^/yr; p = 0.02). On a 2-month regimen, eyes that received more than 12 injections over the 24 month period similarly had significantly smaller areas of cRORA (2.24 vs. 3.87 mm^2^; p < 0.01).

**Table 3:** Presence, location, area, and growth of complete RPE and outer retinal atrophy.

		Baseline			24 Months			
		Eyes	Subfoveal	Area (mm^2^)	Eyes	Subfoveal	Area (mm^2^)	Growth Rate (mm^2^/yr)
		n (%)	n (%)	mean (SD)	n (%)	n (%)	mean (SD)	mean (SD)
All Participants		113/197 (57.36%)	25 (12.69%)	1.71 (3.40)	144/197 (73.10%)	45 (31.25%)	2.93 (3.86)	0.60 (1.69)
Drug Group								
	All 3 Drugs	5/6 (83.33%)	2/5 (40.00%)	1.60 (2.35)	6/6 (100.00%)	3/6 (50.00%)	2.55 (2.82)	0.48 (1.43)
	Avastin	19/27 (70.37%)	6/16 (37.50%)	1.86 (3.17)	23/27 (85.19%)	10/18 (55.56%)	4.21 (4.05)	1.15 (2.25)
	Avastin + Eylea	19/27 (70.37%)	5/16 (31.25%)	2.72 (4.86)	21/27 (77.78%)	4/17 (23.53%)	3.11 (4.09)	0.34 (1.94)
	Avastin + Lucentis	1/3 (33.33%)	N/A	0.03 (0.06)	2/3 (66.67%)	1/2 (50.00%)	3.33 (4.24)	1.65 (2.13)
	Eylea	39/71 (54.93%)	11/36 (30.56%)	1.66 (3.49)	47/71 (66.20%)	15/45 (33.33%)	2.66 (4.21)	0.49 (1.41)
	Eylea + Lucentis	9/16 (56.25%)	1/9 (11.11%)	2.28 (3.86)	11/16 (68.75%)	4/11 (36.36%)	2.93 (3.67)	0.06 (0.95)
	Lucentis	21/47 (44.68%)	0/21 (0.00%)	1.03 (2.19)	34/47 (77.34%)	8/33 (24.24%)	2.53 (3.21)	0.72 (1.75)
		p = 0.16	p = 0.09	p = 0.49	p = 0.14	p = 0.34	p = 0.72	p = 0.34
Injections								
	1–10 total injections	37/62 (59.68%)	7/32 (21.88%)	1.78 (3.54)	44/56 (78.57%)	17/39 (43.59%)	4.02 (4.29)	1.05 (2.05)
	11+ total injections	75/134 (55.97%)	18/67 (26.87%)	1.68 (3.35)	100/131 (76.34%)	28/93 (30.11%)	2.46 (3.57)	0.41 (1.48)
		p = 0.63	p = 0.60	p = 0.85	p = 0.74	p = 0.14	p = 0.01	p = 0.02
	1–12 total injections	52/86 (60.47%)	11/44 (25.00%)	2.05 (3.92)	63/79 (79.75%)	25/57 (43.86%)	3.87 (4.50)	0.84 (2.07)
	13+ total injections	61/111 (54.95%)	15/55 (25.45%)	1.44 (2.92)	81/108 (75.00%)	20/75 (26.67%)	2.24 (3.16)	0.43 (1.34)
		p = 0.44	p = 0.96	p = 0.21	p = 0.45	p = 0.04	p < 0.01	p = 0.10
No Baseline cRORA								
	No Incident cRORA	0/34 (0.00%)	N/A	0.00 (0.00)	34/34 (100.00%)	N/A	N/A	N/A
	Incident cRORA	0/44 (0.00%)	N/A	0.00 (0.00)	44/44 (100.00%)	13/39 (33.33%)	3.31 (3.59)	1.65 (1.79)
		p = N/A	p = N/A	p = N/A	p = N/A	p = N/A	p = N/A	p = N/A

Abbreviations: cRORA, complete RPE and outer retinal atrophy; SD, standard deviation

## Discussion

In this study, the Advanced RPE Analysis software tool was used to automatically identify and quantify progression of cRORA in eyes treated with aflibercept, bevacizumab and/or ranibizumab. Previous studies relied upon manual grading of fundus imaging to track RPE and outer retinal atrophy, included previously treated eyes, or compared only two anti-VEGF agents. The findings presented here contribute to the ongoing debate regarding the comparative efficacy and safety of these three anti-VEGF agents for the treatment of AMD.

The baseline characteristics were similar as those reported in a meta-analysis of the natural history of AMD, as well as recent clinical trials in AMD[26–28]. Patients treated were predominantly in their mid/late-70s with a slight female majority. The mean baseline visual acuity of 0.63 ± 0.52 logMAR (20/85 Snellen equivalent) is in line with the baseline visual acuity seen in the Inhibition of VEGF in Age-related choroidal Neovascularisation (IVAN) and CATT trials. In this study and the ‘discontinuous regimen’ arm of the IVAN trial, patients were given three consecutive monthly injections before transitioning to a pro re nata (PRN) schedule. Patients in the ‘PRN’ arm of the CATT trial were treated as needed from the start of the study enrollment. Average number of injections over two years were 12.9, 13, and 13.1 for this study, the IVAN trial, and the CATT trial, respectively.

Prevalence and incidence of cRORA in this study were relatively high, yet within range of similar studies. At enrollment, 113/197 (57.4%) of study eyes had detectable cRORA, and 44/78 (56.4%) of the eyes without baseline cRORA developed cRORA after 24 months. By comparison, the CATT trial specifically excluded eyes with baseline cRORA and 43/604 (7.1%) of eyes in the IVAN trial had baseline cRORA[27,28]. Retrospective cohort studies noted preexisting cRORA in 18.1% - 59.3% of eyes[13–15,29]. Incident cRORA ranged from 18.3% - 61%[13–15,27–30]. The higher proportion of eyes with atrophy may have partially contributed to the sensitivity of the Advanced RPE Analysis algorithm in identifying small lesions that may have otherwise passed undetected by manual measurement of color fundus photographs. In a cross-sectional study of 50 eyes diagnosed with atrophic AMD, the smallest lesion detected by manual sub-RPE slab measurement was 0.17 mm^2^. From the same sub-RPE slab images, the automated algorithm detected lesions as small as 0.10 mm^2^ –a 41% difference. The mean size of all cRORA lesions detected with the automated algorithm was also significantly smaller at 5.27 mm^2^ versus a mean size of 6.41 mm^2^ for cRORA measured manually.

Several baseline risk factors were significantly associated with the development and growth of cRORA. Understandably, indicators of advanced AMD (e.g. poor baseline visual acuity and presence of cRORA at baseline) strongly correlated with cRORA progression. In the CATT trial, presence of baseline cRORA in the fellow eye increased the risk of two-year cRORA incidence more than twofold (39.5% with cRORA in fellow eye; 16.3% without cRORA in fellow eye). The annual growth rate incrementally increased from 0.31 mm^2^/year in eyes with baseline cRORA area < 1 mm^2^ to 0.47 mm^2^/year in eyes with baseline cRORA area > 5 mm^2^[27,31]. Lois et al. retrospectively reviewed 72 eyes and found the association between baseline atrophy and two-year atrophy to approach significance with skew towards increased odds of cRORA (odds ratio, 2.65; 95% confidence interval, 0.84–8.39; p = 0.10)[14]. Study eyes with a baseline visual acuity of 20/200 or worse had nearly double the risk of cRORA development than eyes with baseline VA between 20/25-40 (hazard ratio, 1.89; 95% confidence interval, 1.07–3.37; p = 0.03)[27].

Baseline drusen, particularly drusen volume, has been found to be an independent predictor of AMD progression and cRORA development[32–34]. On the other hand, some studies such as the AREDS 2 Ancillary SD-OCT Study did not find OCT-detected drusen to significantly associate with new-onset cRORA[35]. The lack of an association between drusen volume with cRORA in the current study is more likely a reflection of a smaller participant enrollment and statistical power rather than a confirmation or refutation of findings published previously.

In the Beaver Dam Eye Study (N = 4756), Rotterdam Study (N = 6411), Blue Mountains Eye Study (N = 3585), and Copenhagen City Heart Study (N = 1000), past history of stroke was determined to not be a risk factor for incident atrophy[36–38]. The current study supports the absence of a significant association between stroke history and atrophic AMD. However, its association with larger cRORA area and faster cRORA growth rate is a new finding not reported by any other study in the literature. Further studies are needed to confirm whether history of stroke is a risk factor for larger and faster growing cRORA lesions.

The two-year results from the CATT trial raised concerns that frequent use of anti-VEGF injections may accelerate cRORA. Eyes that were randomized to monthly injections instead of PRN injections had a 59% increase in the development of new cRORA (p < 0.01)[27]. This finding was replicated in the IVAN trial in which patients on the continuous instead of the discontinuous regimen had significantly higher percentages of eyes with new cRORA (odds ratio 1.47; 95% confidence interval, 1.03–2.11; p = 0.03)[28]. Significant associations between number of anti-VEGF injections and growth rate of cRORA were found in retrospective studies by Young et al. (number of ranibizumab injections, p < 0.01; number of bevacizumab injections, p < 0.01) and Abdelfattah et al. (total number of injections, p = 0.01)[13,15].

Other cohort studies have found conflicting associations between number of anti-VEGF treatment and retinal atrophy[29]. Although Xu et al. found increased odds of new cRORA based on number of injections (p = 0.02), there was no significant association with cRORA growth. Conversely, Lois et al. found a significant correlation between total number of treatment and rate of cRORA progression (p = 0.02), but not with cRORA incidence[14]. However, no previous study has ever reported an inverse relationship. As noted earlier, eyes enrolled in this study were placed on treat-and-extend regimens similar to the PRN cohort or discontinuous regimen cohort of the CATT and IVAN trials, respectively. Whereas clinical trials have shown that eyes treated continuously (mean 23 injections) have significantly more cRORA than eyes treated discontinuously (mean 13 injections), stratifying treat-and-extend participants has further revealed that eyes on the lower end of injection frequency (mean ≤10 total injections) also fare poorly in long-term cRORA outcomes.

By most accounts, aflibercept, bevacizumab, and ranibizumab have been shown to have comparable effects in improving or stabilizing visual acuity in eyes with nAMD. A meta-analysis of 5,225 patients with nAMD found that monthly injections of bevacizumab is non-inferior to monthly injections of ranibizumab. Visual endpoints for aflibercept injections every 8 weeks has also been found to perform similarly with monthly ranibizumab injections[39]. However, their individual effects on cRORA remain inconclusive. Two-year outcomes from the CATT trial found ranibizumab-treated eyes to develop cRORA at higher rates than bevacizumab-treated eyes (adjusted hazard ratio, 1.43; 95% confidence interval, 1.06–1.93), whereas cRORA development did not differ by drug group in the IVAN trial[27,28]. Neither of these clinical trials had compared cRORA following treatment with aflibercept. Retrospective studies that included aflibercept-treated eyes were limited either by the low proportion of recruited patients who received only aflibercept, the low median number of total aflibercept injections, or did not evaluate cRORA outcomes based on drug group[13,29,30,40]. In the current study, no significant difference in the incidence or growth of cRORA was found between monotherapy or combination therapy with aflibercept, bevacizumab, or ranibizumab.

Strengths of this study include the use of two-year long-term cRORA outcomes as well as the inclusion of eyes treated with any of the three anti-VEGF agents. Use of the validated Advanced RPE Analysis tool also reduces the effect of inter-grader variation associated with manual fundus measurement, and enhances study replicability for future studies at other clinical sites. Several limitations should be considered due to the retrospective design of this study. The treatment schedule and choice of drug was left to the discretion of the individual patient and treating physician. Nonetheless, these treatment decisions have value by reflecting the true preferences of physicians and patients in a real clinical practice. Despite being provided return dates after each clinic visit, patients had inconsistent adherence with recommended injection schedules. This was taken into account by evaluating the association of cRORA with the number of injections. Lastly, the number of enrolled eyes in total and for each drug group was smaller than that of the CATT and IVAN trials. A larger study with increased power to detect a potential difference between anti-VEGF agents is warranted.

## Conclusion

In a treat-and-extend regimen, incidence and growth of cRORA was not associated with choice of anti-VEGF treatment for nAMD. This is the first study to report an inverse association between number of anti-VEGF injections and cRORA development. Future investigation with randomized, prospective studies are needed to evaluate whether cRORA progression differs between aflibercept, bevacizumab, or ranibizumab.
